# Removal of Arsenic(III) from Aqueous Solution Using Metal Organic Framework-Graphene Oxide Nanocomposite

**DOI:** 10.3390/nano8121062

**Published:** 2018-12-16

**Authors:** Tonoy Chowdhury, Lei Zhang, Junqing Zhang, Srijan Aggarwal

**Affiliations:** 1 Department of Mechanical Engineering, P.O. Box 755905, University of Alaska Fairbanks, Fairbanks, AK 99775, USA; tchowdhury@alaska.edu; 2 Department of Civil & Environmental Engineering, P.O. Box 755900, University of Alaska Fairbanks, Fairbanks, AK 99775, USA; saggarwal@alaska.edu

**Keywords:** nanocomposite, graphene oxide, metal-organic framework, heavy metal, adsorption

## Abstract

MIL-53(Al)-graphene oxide (GO) nanocomposites of different GO to MIL-53(Al) mass ratios (1% to 25% GO) were synthesized and tested for removal of arsenite (As(III)), which is a well-known groundwater contaminant. The properties of MIL-53(Al)-GO nanocomposites were characterized using X-ray Diffraction (XRD), Fourier Transform Infrared (FT-IR) Spectroscopy, Brunauer-Emmett-Teller (BET) surface area measurements, and Scanning Electron Microscopy (SEM). Batch experiments were performed on MIL-53(Al)-GO nanocomposites for As(III) adsorption in aqueous solutions to investigate adsorption kinetics and isotherm behavior under varying environmental conditions. The effects of solution pH (2 to 11), initial As(III) concentrations (10–110 mg/L), adsorbent dosage (0.2–3.0 g/L), and temperature (298–318 K) on As(III) adsorption were investigated. MIL-53(Al)-GO nanocomposites showed higher adsorption of As(III) than pristine MIL-53(Al) and GO individually. As (III) removal was optimized at a ratio of 3% GO in the MIL-53(Al)-GO nanocomposite, with an adsorption capacity of 65 mg/g. The adsorption kinetics and isotherms followed pseudo-second-order and Langmuir isotherm models, respectively. Overall, these results suggest that MIL-53(Al)-GO nanocomposite holds a significant promise for use in the remediation of As (III) from groundwater and other aqueous solutions.

## 1. Introduction

Water is contaminated continuously by waste products from various sources such as textile, leather, food processing, mining, oil, agricultural, and pharmaceutical industries [1]. This contaminated water consists of heavy metals like Pb(II), Hg(II), As(III), and Cd(II) ions [2,3,4], different types of organic and inorganic dyes [5,6,7], detergent and oil [8], pharmaceutical and personal care products [9,10,11,12,13], nitrogen containing compounds (NCCs) [14], sulfur containing compounds [1], and many more that are highly toxic for living organisms [15,16].

Among the heavy metal species, both As(III) and As(V) are considered to be highly toxic pollutants in water [17]. Their natural presence in soil, water, rocks, and food makes it even more hazardous [18]. Arsenic in ground waters has severe impacts for human health and well-being [19]. Exposure to inorganic arsenic for a long time can cause lung, bladder, kidney and skin cancer, and pigmentation changes [20]. Moreover, nausea, dryness of mouth and gastro-intestinal symptoms can also be observed in case of acute and chronic arsenic poisoning [19].

To remove heavy metal ions, various physical and chemical methods are used, which include, but are not limited to, adsorption, solvent extraction [21], ion exchange [22], precipitation [23], filtration [24,25], and photocatalytic degradation [26,27]. Adsorption processes with different cost effective porous adsorbents draw much attention due to their high removal capacity with selectivity, ease and simplicity of operation, and low generation of harmful byproducts [28]. Porous activated carbons, carbon nanotubes, zeolites, and bioadsorbents are widely used as adsorbents for heavy metal removal [1,29,30]. However, practical applications of these materials are limited by their low adsorption capacities, low efficiencies, or high cost.

In recent years, nanomaterials have shown excellent potential for enhanced adsorption of heavy metals compared to traditional adsorbents, due to their superior properties. Graphene oxide (GO), a derivative of graphene sheets with oxygen-containing groups on the surfaces, has been proven to possess extremely high adsorption capacity for removing heavy metals from contaminated water [31,32,33]. Metal-organic frameworks (MOFs), a newly emerged class of nanoporous materials that are organic-inorganic hybrids constructed from metal ion nodes linked by organic linkers to form a three-dimensional (3D) crystal lattice structure [34], have received considerable attention due to their ultra-high surface areas, low densities, diverse topologies, and tunable pore sizes. These excellent properties of MOFs make them widely applicable in water treatment, gas storage, catalysis, and drug delivery [1,2,7,35,36,37,38]. Different heavy metal ions like As(V), Cd(II), Co(II), Cr(VI), Ni(II), Pb(II), and Zn(II) have been reported to be adsorbed onto MOF and its composites [1,2]. Incorporating GO into MOFs to create a nanocomposite is an effective and feasible approach to removal heavy metal ions. MOF-GO composites have been reported to be great adsorbents for organic compounds like dyes and NCCs [39,40,41], but are rarely reported for heavy metal adsorption [42]. This motivates us to synthesize and test MOF-GO composites for heavy metal removal.

In this study, we synthesized MOF-GO nanocomposites using an aluminum-based MOF MIL-53(Al) (AlC_8_H_5_O_5_) and GO at varying ratios; and investigated the as-synthesized nanocomposites for As(III) adsorption under different solution conditions. MIL-53(Al) is built by the interconnection of infinite trans chains of corner-sharing AlO_4_(OH)_2_ octahedra with 1,4-benzenedicarboxylate ligands. MIL-53(Al) was selected in this study because it has low density and high surface area, and more importantly, it is highly stable and resistant to hydrolysis in aqueous solutions [43]. Our results demonstrate that the textural properties of MIL-53(Al)-GO nanocomposite can be tailored by tuning the mass ratio of GO to MIL-53(Al); more importantly, MIL-53(Al)-GO exhibits higher As(III) adsorption capacity compared to individual moieties. The effects of solution pH, initial metal ion concentration, adsorbent dosage, and temperature on As(III) adsorption were investigated in batch studies to understand the kinetic and isotherm behavior of the MIL-53(Al)-GO nanocomposite in varying environmental conditions.

## 2. Materials and Methods

### 2.1. Materials

Aluminum(III) nitrate nonahydrate (Al(NO_3_)_3_·9H_2_O), terephthalic acid (H_2_BDC), and *N*,*N*-dimethylformamide (DMF) were used to prepare MIL-53(Al). Graphene oxide was synthesized using 99% sulfuric acid (H_2_SO_4_), graphite, potassium permanganate (KMnO_4_), and 30% hydrogen peroxide (H_2_O_2_). Arsenic ICP standard solution (10,000 ppm As(III) in 5% HNO_3_) was purchased from Ricca Chemical Company (Arlington, TX, USA) and other chemicals were purchased from Sigma-Aldrich (St. Louis, MO, USA). Methanol (CH_3_OH) used to wash MIL-53(Al) was purchased from VWR scientific (West Chester, PA, USA). All reagents and solvents were analytic grade and used as received. Deionized water (DI) was produced using a Thermo Scientific Barnstead NANO pure purifying system (18.2 MΩ·cm). Micro porous (4–7 µm) filter papers were used to separate the adsorbent from the As(III) solutions for atomic spectroscopy analyses.

### 2.2. Synthesis of GO

Graphene oxide was prepared using modified Hummers method [44]. 0.5 g graphite powder, 0.5 g of NaNO_3_ and 23 mL of H_2_SO_4_ (99%) were mixed by stirring in a glass beaker placed in an ice bath for 4 h. Three grams of KMnO_4_ was slowly added to the mixture. The mixture was always kept below 20 °C to avoid overheating and explosion. After a few minutes, the ice bath was removed and the temperature of the mixture was increased up to 35 °C for 1 h with continued stirring. Forty-six mL of DI water was slowly added to the mixture and temperature was elevated to 95 °C. Aluminum foil was used to cover the beaker to avoid the mixture boiling off. After 2 h of heating, the mixture was cooled down to room temperature. Then, 100 mL of DI water was added and stirred followed by adding 10 mL of 30% H_2_O_2_. The reaction mixture was further stirred for about 30 min. Finally, the brownish product was washed three times with DI water, centrifuged (8000 rpm, 10 min), and freeze dried for 48 h to obtain GO powder.

### 2.3. Synthesis of MIL-53(Al)

MIL-53(Al) was synthesized using a method as reported by Ricco et al. [45]. 0.788 g of Al(NO_3_)_3_·9H_2_O (2.1 mmol) and 0.518 g of H_2_BDC (3.12 mmol) were mixed in 30 mL of DMF. The mixture was put in a 150 mL stainless steel autoclave with a Teflon inset at 130 °C for 72 h. A white gel was obtained and separated by centrifugation (8000 rpm, 10 min). The sample was then washed three times with 30 mL methanol and centrifuged as above followed by air dry at 100 °C overnight. The white product was immersed in methanol (30 mL) for 24 h, washed and centrifuged three times with methanol, following the procedures as above. Finally, the sample was dried overnight under vacuum at 110 °C.

### 2.4. Synthesis of MIL-53(Al)-GO Nanocomposites

MIL-53(Al)-GO nanocomposite was synthesized by dispersing a certain amount of GO powder in 30 mL DMF solution along with Al(NO_3_)_3_·9H_2_O and H_2_BDC. The solution was sonicated for 10 min to obtain a homogeneous suspension and then subjected to the same synthesis procedure of MIL-53(Al). MIL-53(Al)-GO nanocomposite was denoted as n% MIL-53(Al)-GO, where n is the mass ratio of GO to MIL-53(Al). 1%, 2%, 3%, 5%, 10%, 15%, and 25% MIL-53(Al)-GO nanocomposites were synthesized.

### 2.5. Adsorbent Characterization

Crystal structures of MIL-53(Al)-GO, MIL-53(Al) and GO were examined using a Rigaku MiniFlex II X-ray diffractometer (Rigaku Americas Corporation, The Woodlands, TX, USA). A Scientific Nicolet 6700 Fourier Transform Infrared (FT-IR) Spectroscopy (Thermo Scientific, Waltham, MA, USA) was used to characterize the functional groups in MIL-53(Al)-GO, MIL-53(Al) and GO. To examine the morphologies of the composites and parent materials, a JEOL JXA-8530F Scanning Electron Microprobe (JEOL USA, Inc., Peabody, MA, USA) was used. Surface areas of MIL-53(Al)-GO, MIL-53(Al) and GO were measured by nitrogen adsorption at 77 K, the samples were degassed at 110 °C for 12 h before measurement and surface areas were calculated using the Brunauer-Emmett-Teller (BET) model. Zeta potentials of MIL-53(Al)-GO at pH = 4–11 (adjusted using 0.1 M NaOH or 0.1 M HNO_3_) were determined using a BIC ZetaPlus zeta potential meter (Brookhaven Instruments Corporation, Holtsville, NY, USA).

### 2.6. Adsorption Experiments

Batch experiments were carried out to study the As(III) adsorption kinetics and thermodynamics on MIL-53(Al)-GO nanocomposites and the factors affecting adsorption. All the tests were performed in triplicates. Prior to the experiment, stock aqueous solutions of As(III) of different initial concentrations (10–120 mg/L) were adjusted to pH 6.0 using 0.1 M NaOH or 0.1 M HNO_3_. The adsorbent was heated overnight under vacuum at 110 °C to remove any moisture.

*Kinetic Studies*. For adsorption kinetics study, 5 mg of adsorbent was added to 25 mL As(III) solution containing As(III) at a concentration of 50 mg/L. The mixture was sonicated in a water bath sonicator (Vevor Digital Ultrasonic Cleaner, PS-40A, Shanghai, China) for 10 min to obtain a homogeneous solution and then transferred into 250 mL polypropylene bottles. The mixture was shaken in a gyratory shaker (Orbital Shaker, VWR S-500, Radnor, PA, USA) at 175 rpm and 298 K. At predetermined time intervals (from 1 min to 24 h; or until the concentrations reached equilibrium), polypropylene bottles were removed from the shaker. The adsorbent was then separated from the reaction mixture by centrifugation (8000 rpm, 8 min) and subsequent filtration.

*Isotherm Studies*. To obtain adsorption isotherms, 5 mg of adsorbent (MIL-53(Al), GO, and 3% MIL-53(Al)-GO) was added to 25 mL of As(III) solution with As (III) concentration varying from 10 to 110 mg/L, and this was repeated at three different temperatures (298, 308 and 318 K). The effect of pH on As(III) adsorption was examined by mixing 5 mg of 3% MIL-53(Al)-GO) with 25 mL of As(III) solution (50 mg/L) at 298 K under 175 rpm. The initial pH of As(III) solution in the range of 4 to 11 was adjusted using 0.1 M NaOH or 0.1 M HNO_3_.

The effect of initial ion concentration on As(III) adsorption was examined by agitating 5 mg of 3% MIL-53(Al)-GO) with 25 mL of As(III) solution (10–110 mg/L) under 175 rpm at 298 K. Adsorbent (3% MIL-53(Al)-GO) concentration was varied from 0.2 to 3 g/L, and added in 50 mg/L As(III) solution to examine the effect of adsorbent dosage on As(III) adsorption.

For all the above described experiments, concentration of As(III) in the solution was determined using a microwave induced plasma interfaced atomic emission spectrophotometer (Agilent Technologies, 4200 MP-AES, Santa Clara, CA, USA). The mass of As(III) ions adsorbed per unit mass of adsorbent at equilibrium is defined as the equilibrium adsorption capacity (*q_e_*), which was calculated using Equation (1):

(1) $$q_{e}=\frac{C_{0}-C_{e}}{M}V$$

where *q_e_* (mg/g) is the equilibrium adsorption capacity, *C*_0_ (mg/L) and *C_e_* (mg/L) are the initial and equilibrium concentration of As(III), respectively, *V* (mL) is the volume of As(III) solution and *M* (mg) is the mass of adsorbent. Removal efficiency of As(III) was calculated using Equation (2).

(2) $$\mathrm{Removal}\;\mathrm{Efficiency}\;=\frac{C_{0}-C_{e}}{C_{0}}\;\times 100\%$$

## 3. Results and Discussion

### 3.1. Nanomaterial Characterization

X-ray diffraction (XRD) patterns of GO, MIL-53(Al), and MIL-53(Al)-GO nanocomposites are shown in Figure S1a. The characteristic peaks at 2*θ* = 8.8°, 15.25° and 17.75° for MIL-53(Al) and 2*θ* = 11° for GO confirmed their crystal structures which were in agreement with the previously reported work [46,47]. MIL-53(Al)-GO nanocomposites showed diffraction patterns similar to the pure MIL-53(Al). It is worth noting that the diffraction patterns of MIL-53(Al)-GO nanocomposites are right-shifted with regard to that of the pristine MIL-53(Al). According to Bragg’s law, this indicates that the constant parameters of MIL-53(Al)-GO nanocomposites become smaller compared to that of MIL-53(Al), which is due to the incorporation of GO sheets. In addition, no diffraction peak for GO was observed in the nanocomposites, confirming that GO is coordinated with the MIL-53(Al) [48,49]. It was observed that the crystallinity of MIL-53(Al)-GO was reduced when GO content in MIL-53(Al) was increased, which is probably due to the MIL-53(Al) cage separation and completely separated GO sheets [14]. FT-IR spectra of GO, MIL-53(Al) and MIL-53(Al)-GO are shown in Figure S1b. MIL-53(Al) exhibited vibration bands at 1400–1700 cm^−1^ for the carboxylic functional group [50]. Characteristic peaks of GO were observed at 3417 cm^−1^ for O–H stretching, 1623 cm^−1^ for C=C stretching, 1722 and 1407 cm^−1^ for carboxyl group stretching and 1230 and 983 cm^−1^ for C–O stretching [47]. All of the characteristic peaks of MIL-53(Al) and GO were observed in the spectra of MIL-53(Al)-GO nanocomposites.

**Figure 1:**
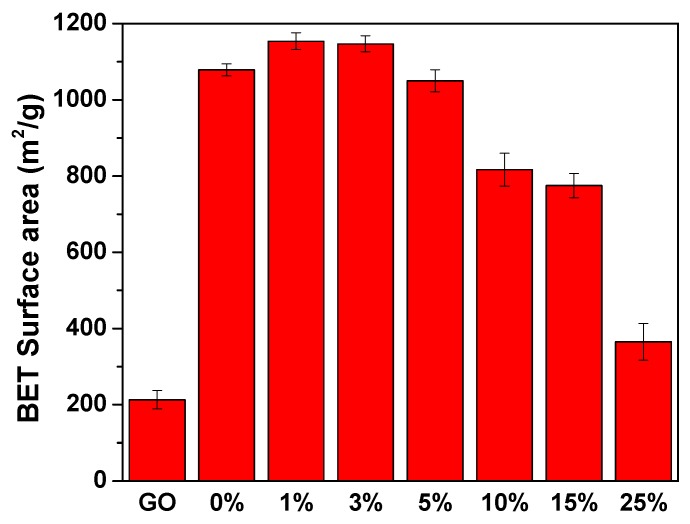
BET surface area of GO, MIL-53(Al) and MIL-53(Al)-GO nanocomposites (n% = MIL-53(Al)-GO nanocomposite with n% mass ratio of GO to MIL-53(Al); 0% = MIL-53(Al)).

BET surface areas of GO, MIL-53(Al) and MIL-53(Al)-GO nanocomposites are shown in Figure 1. It shows that surface areas of MIL-53(Al)-GO increased as the GO content in the composite increased. The surface area increase of MIL-53(Al)-GO nanocomposites might have resulted from (a) the separation of cages of MIL-53(Al) due to the intersection of GO layers and (b) attachment of epoxy and hydroxyl functional groups of GO layers with MIL-53(Al) [14]. A reduction in surface area was observed when the mass ratio of GO to MIL-53(Al) was higher than 3%. A high content of GO was not suitable for composite formation due to the limited capability of MIL-53(Al) to integrate with the GO sheets in some orientations. This indicates that the textual properties of MIL-53(Al)-GO nanocomposite can be tailored by tuning the content of GO, which is associated with the intersection degree of GO layers with MIL-53(Al).

The morphologies of GO, MIL-53(Al) and MIL-53(Al)-GO nanocomposites are shown in Figure S2. GO shows a stacked and crumpled morphology (Figure S2a). In Figure S2b, aggregation of MIL-53(Al) particles can be clearly observed. The morphology of MIL-53(Al) appeared different after composited with GO (Figure S2b–f). As the GO content increased, sphere-shaped particles of MIL-53(Al)-GO nanocomposites were observed, which were dispersed homogenously. During the synthesis of MIL-53(Al)-GO, coordination of the oxygen–containing groups from GO with the metal sites and ligands of MIL-53(Al) provided homogeneous nucleation nodes for the formation of MIL-53(Al)-GO, which led to the homogeneous assembly of MIL-53(Al)-GO particles [48,49].

### 3.2. Adsorption Kinetics

**Figure 2:**
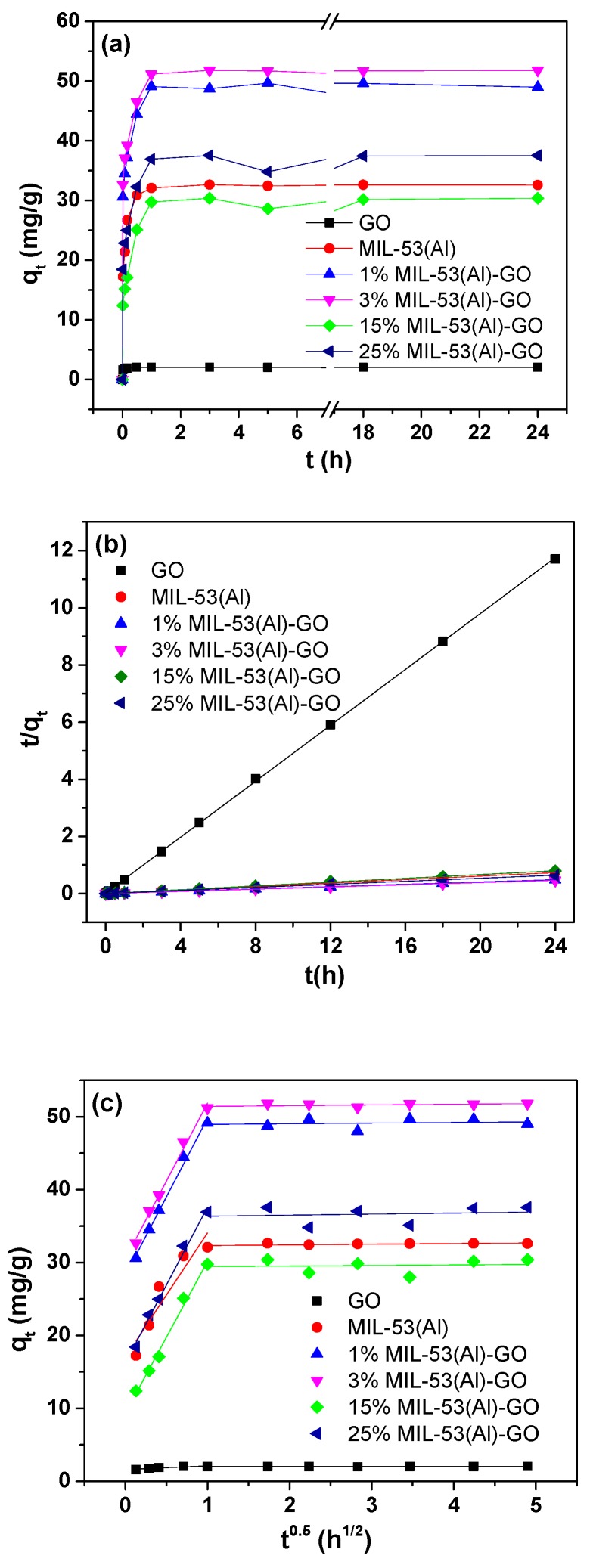
(**a**) Adsorption capacity of As(III) as a function of contact time, (**b**) pseudo-second-order plots, and (**c**) intra-particle diffusion for As(III) adsorption on GO, MIL-53(Al), and MIL-53(Al)-GO nanocomposites.

Figure 2a shows the adsorption capacity of As(III) as a function of contact time on GO, MIL-53(Al) and MIL-53(Al)-GO nanocomposites. As(III) adsorption rate decreased with time until reaching adsorption equilibrium for all the samples. All the MIL-53(Al)-GO nanocomposites exhibited fast adsorption rates and reached an equilibrium state in less than one hour, which indicates that MIL-53(Al)-GO is an efficient adsorbent. Highest As(III) adsorption was found for 3% MIL-53(Al)-GO (51.8 mg/g), which was 58% and 766% higher than those with pristine MIL-53(Al) and GO, respectively. Pseudo-first-order and pseudo-second-order equations were used to explain the adsorption kinetics of As(III) ions. Pseudo-first-order kinetics [6] is defined by Equation (3):

(3) $$log\left(q_{e}-q_{t}\right)=logq_{e}-\frac{tk_{1}}{2.303}$$

where *q_e_* and *q_t_* (mg/g) are the mass of As(III) ions adsorbed per unit mass of adsorbent at equilibrium and time *t* (h), respectively, and *k*_1_ (1/h) is the rate constant for a pseudo-first-order reaction. Values of *k*_1_ were calculated from plots of *log*(*q_e_* − *q_t_*) vs. *t* for As(III) adsorption on adsorbents tested. Pseudo-second-order kinetics [6] is expressed as Equation (4) below:

(4) $$\frac{t}{q_{t}}=\frac{1}{k_{2}q_{e}^{2}}+\frac{t}{q_{e}}$$

where *k*_2_ (g/mg·h^−1^) is the rate constant of the pseudo-second-order reaction. Values of *k*_2_ were calculated from plots of *t*/*q_t_* vs. *t* for As(III) adsorption on the adsorbents tested.

Table 1 summarizes the pseudo-first-order and pseudo-second-order kinetic parameters of As(III) adsorption onto adsorbents. By comparing with the correlation coefficients (*R*^2^) of the pseudo-first-order and pseudo-second-order models, it was concluded that the pseudo-second-order kinetic model fit the adsorption data of all the samples better than the pseudo-first-order model. The calculated values of equilibrium adsorption capacity of As(III) (*q_e,cal_*) from the pseudo-second-order kinetic model were very close to experimental values (*q_e,exp_*). The pseudo-second-order plots are shown in Figure 2b.

To further understand the As(III) adsorption kinetics, the intra-particle diffusion model [6] was used to analyze the data:

(5) $$q_{t}=k_{id}t^{1/2}+C_{i}$$

where *i* is the number of piecewise linearity, *k_id_* is the intra-particle diffusion rate constant (mg/(g·h^1/2^)), and *C_i_* is the intercept related to the boundary layer thickness. If there is only one good linear fit section to the data of the adsorption process, it implies that the whole adsorption process is dominated by intra-particle diffusion (*i* = 1 and *C* = 0), and the intra-particle diffusion is the only rate-limiting step. Otherwise, the larger the intercept, the greater degree of film diffusion sorption involved in rate controlling [51]. As shown in Figure 2c, there are two linear sections for As(III) adsorption data in this study, which implies a two-step adsorption process. The fitting parameters of the intra-particle diffusion model for each step are listed in Table 1. Intra-particle diffusion was found to be the rate-controlling step after comparing the values of correlation coefficients, (*R*_1_)^2^ and (*R*_2_)^2^. At the beginning of adsorption (the first linear section in Figure 2c), intra-particle diffusion controlled the diffusion of As(III) ions from the solution to MIL-53(Al)-GO nanocomposite, which might also be accompanied by film diffusion. In the end (the linear section with a large slope in Figure 2c), the adsorption reached equilibrium and the film diffusion dominated the As(III) adsorption.

### 3.3. Adsorption Isotherms

**Figure 3:**
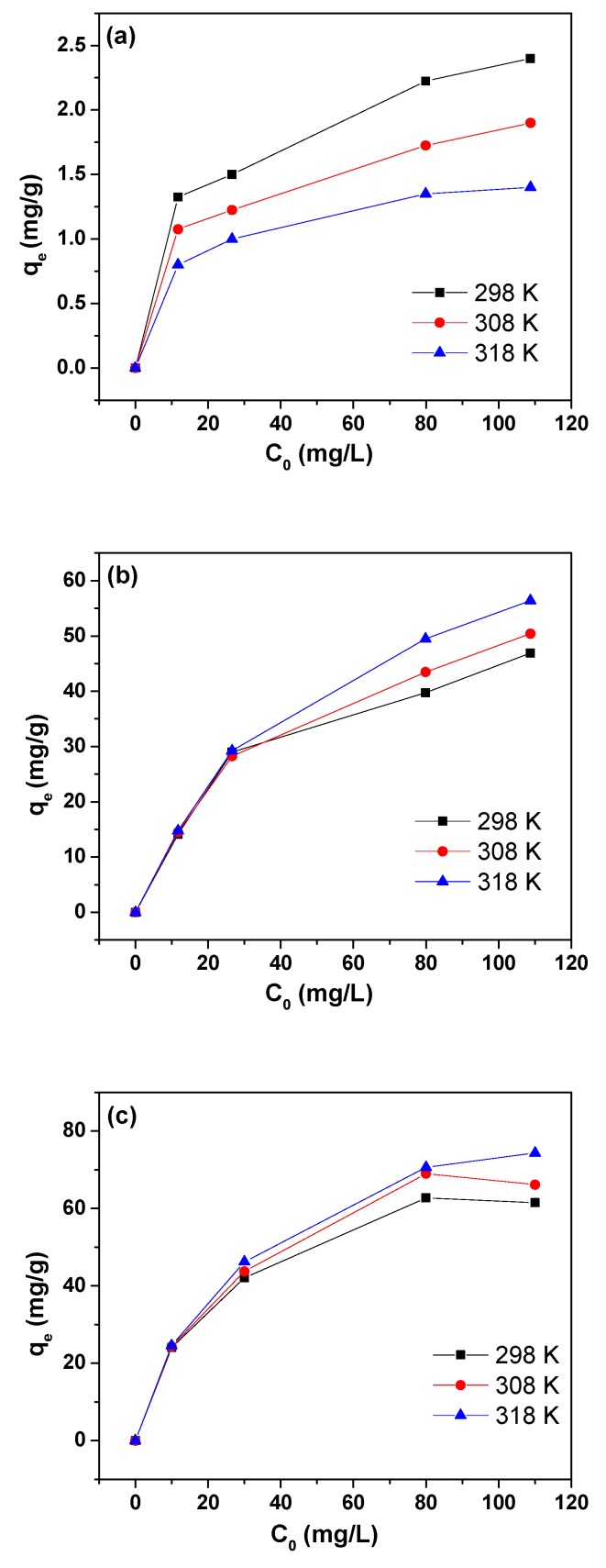

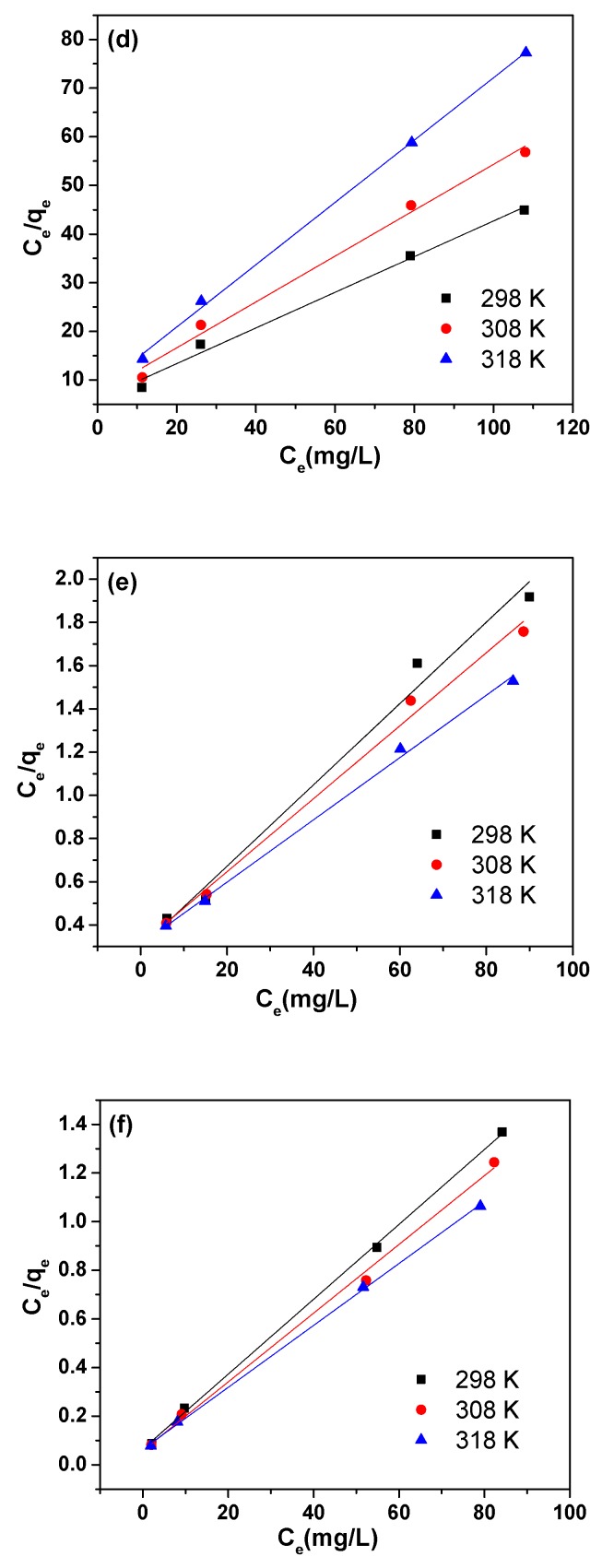
As(III) adsorption by (**a**) GO, (**b**) MIL-53(Al), and (**c**) 3% MIL-53(Al)-GO at different temperatures; Langmuir isotherm models for As(III) adsorption by (**d**) GO, (**e**) MIL-53(Al), and (**f**) 3% MIL-53(Al)-GO.

The adsorption isotherms for GO, MIL-53(Al), and 3% MIL-53(Al)-GO, obtained at different temperatures, are shown in Figure 3a–c. The equilibrium data were analyzed using the Langmuir and Freundlich isotherm models. The Langmuir model assumes a surface with homogeneous binding sites, equivalent adsorption energy, and no interaction between adsorbed species [52]. According to the Langmuir isotherm model, adsorption takes place at specific homogeneous sites on a sorbent and the linear form [48] can be written as

(6) $$\frac{C_{e}}{q_{e}}=\frac{C_{e}}{q_{max}}+\frac{1}{q_{max}K_{L}}$$

Freundlich isotherm model assumes a heterogeneous adsorption surface and active sites with different energy, and the Freundlich isotherm model [48] is given in linear form as

(7) $$lnq_{e}=lnK_{F}+\frac{1}{n}lnC_{e}$$

where *C_e_* (mg/L) is the equilibrium concentration of As(III), *q_e_* and *q_max_* (mg/g) are the equilibrium adsorption capacity and the maximum adsorption capacity of As(III) ions, respectively, and *K_L_* (L/mg) is the Langmuir constant representing the degree of sorption affinity the adsorbate has to the adsorbent. *K_F_* [(mg/g)·(L/mg)^1/*n*^] and 1/*n* are Freundlich constants that denote the adsorption capacity and the adsorption intensity, respectively. 

Isotherm parameters of Langmuir and Freundlich models for the As(III) adsorption are listed in Table 2. The *R*^2^ values for the Langmuir model were higher than those for the Freundlich model, which suggests that the Langmuir model could better describe As(III) adsorption than the Freundlich model. This also indicated that As(III) adsorption was directed by monolayer adsorption on a homogenous surface of the adsorbents tested in this work. The plots showing linear relation between *C_e_*/*q_e_* and *C_e_* of Langmuir model are presented in Figure 3d–f. The Langmuir constant, *K_L_*, for 3% MIL-53(Al)-GO nanocomposite was much higher than that of MIL-53(Al) and GO, indicating a much stronger sorption affinity of 3% MIL-53(Al)-GO nanocomposite to As(III). The maximum adsorption capacity (*q_max_*) values for 3% MIL-53(Al)-GO nanocomposite were much higher than those for MIL-53(Al) and GO at different temperatures. Moreover, the maximum adsorption capacity of 3% MIL-53(Al)-GO was found higher than that of many adsorbents reported [17,53,54,55,56,57], as shown in Table S1. This suggests that MIL-53(Al)-GO nanocomposite may be a promising adsorbent in the application of arsenic removal from groundwater and aqueous solutions in general.

To further understand the feature of Langmuir isotherm, a separation factor (*R_L_*) [48] was calculated using Equation (8):

(8) $$R_{L}=\frac{1}{1+K_{L}C_{0}}$$

where *K_L_* (L/mg) is the Langmuir constant and *C*_0_ (mg/L) is the initial As(III) concentration.

The value of *R_L_* indicates the favorability of the Langmuir isotherm, where *R_L_* > 1 indicates unfavorable, 0 < *R_L_* < 1 indicates favorable, *R_L_* = 1 implies Langmuir isotherm is linear, and *R_L_* = 0 implies Langmuir isotherm is irreversible. The calculated *R_L_* values for As(III) adsorption on GO, MIL-53(Al) and 3% MIL-53(Al)-GO are listed in Table 2. All the *R_L_* values were between 0 and 1, indicating that the Langmuir isotherm was favorable for the adsorption of As(III) on all the specimens. In particular, the smallest *R_L_* values were recorded for 3% MIL-53(Al)-GO, indicating a more favorable adsorption process for As(III) adsorption onto the 3% MIL-53(Al)-GO nanocomposite.

To examine the inherent energy changes related to adsorption, thermodynamic analysis was carried out. Gibbs free energy change, $\Delta G^{0}$ (kJ/mol), in the process of As(III) adsorption is expressed in Equation (9) [47]:

(9) $$\Delta G^{0}=-RT\;lnK_{0}$$

where *T* (K) is the temperature, *R* (=8.314 J/mol·K) is the universal gas constant, and *K*_0_ is the thermodynamic equilibrium constant. *K*_0_ was determined from the intersection of *ln*(*q_e_*/*C_e_*) vs. *q_e_* plot. The enthalpy ($\Delta H^{0}$) and entropy ($\Delta S^{0}$) were calculated from the slope and intersect of the Van’t Hoff plots, respectively. The Van’t Hoff equation is given in Equation (10) [58]:

(10) $$\Delta G^{0}=\Delta H^{0}-T\Delta S^{0}$$

Table S2 lists the calculated adsorption thermodynamic parameters. The negative values of $\Delta G^{0}$ indicate that the adsorption of As(III) ions was spontaneous, and that the interaction of As(III) ions with MIL-53(Al) and 3% MIL-53(Al)-GO was very strong in the temperature range evaluated. However, this was not the case for GO. Moreover, it is noted from Table 2 that the maximum adsorption capacity of As(III) onto GO was very low. It is known that the surface of GO sheets is negatively charged. As(III) is stable at pH < 9.2 in the form of neutral H_3_AsO_3_, while there exists stable negatively charged H_2_As$O_{3}^{-},$ HAs$O_{3}^{2-}$ and As$O_{3}^{3-}$ species in the pH range of 9–12, 12–13, and 13–14, respectively [59]. The electrostatic repulsion between As(III) anions and the GO surface increased with increasing pH value, and this might be the reason for the low adsorption of As(III) onto GO and the positive value of $\Delta G^{0}$ for adsorption of As(III) on GO. Additionally, it is noted that the $\Delta G^{0}$ values of 3% MIL-53(Al)-GO are more negative than those of MIL-53(Al), indicating that the effective sites in 3% MIL-53(Al)-GO are more active than the ones on MIL-53(Al). The positive value of $\Delta H^{0}$ indicates that As(III) adsorption on MIL-53(Al) and 3% MIL-53(Al)-GO was endothermic in nature, and a higher temperature was favorable for As(III) adsorption. This is in agreement with the results of temperature effects. The positive value of $\Delta S^{0}$ specifies a decrease in the order at the interface between the As (III) solution and MIL-53(Al) as well as 3% MIL-53(Al)-GO during the As(III) adsorption process [60].

### 3.4. Effect of pH on As(III) Adsorption

**Figure 4:**
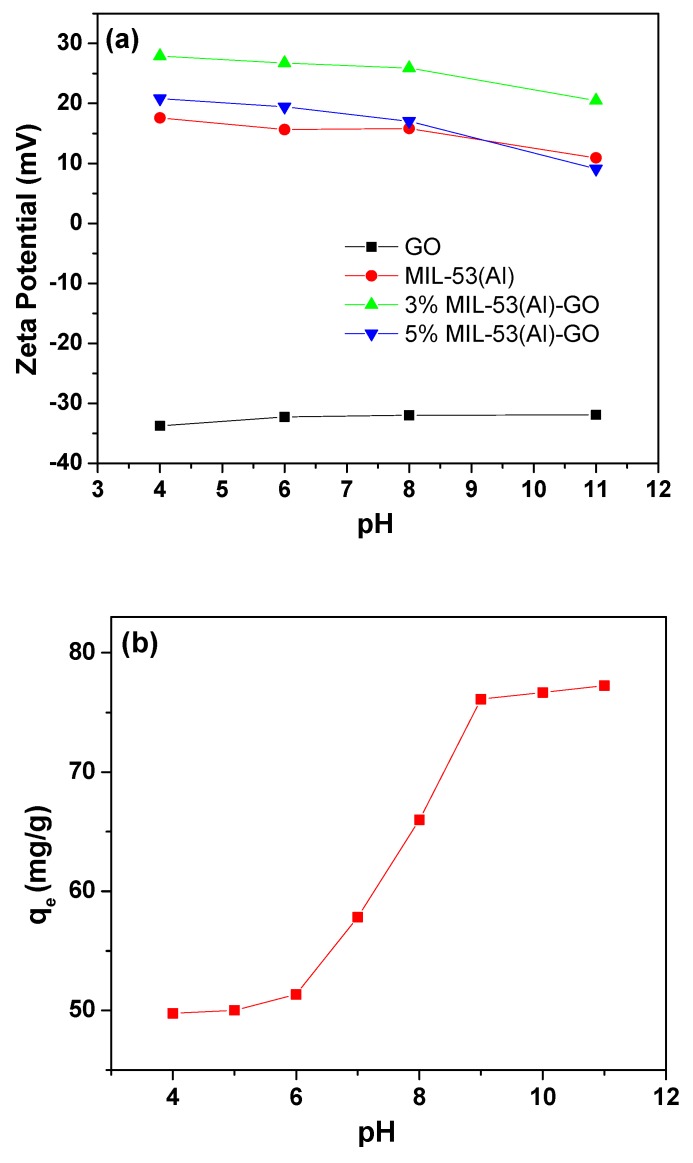
(**a**) Zeta potential of GO, MIL-53(Al) and MIL-53(Al)-GO nanocomposites and (**b**) effect of pH on As(III) adsorption onto 3% MIL-53(Al)-GO (*C*_0_ = 50 mg/L, *M*/*V* = 0.4 g/L, and *T* = 298 K).

The effect of pH on the adsorption of As(III) ions onto 3% MIL-53(Al)-GO is shown in Figure 4. The maximum As(III) adsorption was observed in the pH range of 9–11, and As(III) removal sharply decreased beyond this range. Similar results have been reported in prior studies regarding As(III) adsorption [61,62]. As shown in Figure 4a, the surface potential of MIL-53(Al) and MIL-53(Al)-GO nanocomposites is always positive in the pH range of 4 to 11, which is beneficial to the adsorption of negatively charged pollutants. As mentioned earlier, As(III) is stable at pH < 9.2 as neutral H_3_AsO_3_, while there exists stable negatively charged H_2_As$O_{3}^{-}$, HAs$O_{3}^{2-}$ and As$O_{3}^{3-}$ species in the pH range of 9–12, 12–13, and 13–14, respectively [59]. The amount of negative charge of arsenate increases upon the pH increases from 9 to 13, which is favorable to electrostatic attraction [63,64]. Therefore, electrostatic attraction between the positively charged 3% MIL-53(Al)-GO and negatively charged arsenic species is the key factor dominating the high adsorption of As(III) in the range of 9 < pH < 11. In addition, it is noted that the surface potential of GO is always negative in the pH range of 4 to 11, which implies the electrostatic repulsion between arsenic species and the negatively charged GO. This explains why GO showed a low As(III) adsorption capacity and a positive value of Gibbs free energy change ($\Delta G^{0}$) in As(III) adsorption. In Figure 4a, it is observed that the surface potential of 3% MIL-53(Al)-GO is more positive than that of MIL-53(Al) and 5% MIL-53(Al)-GO at a given pH value, indicating that As(III) adsorption is more favorable on 3% MIL-53(Al)-GO. This is in agreement with the results of As(III) adsorption kinetics and isotherms.

### 3.5. Effect of Initial As(III) Concentration on As(III) Adsorption

**Figure 5:**
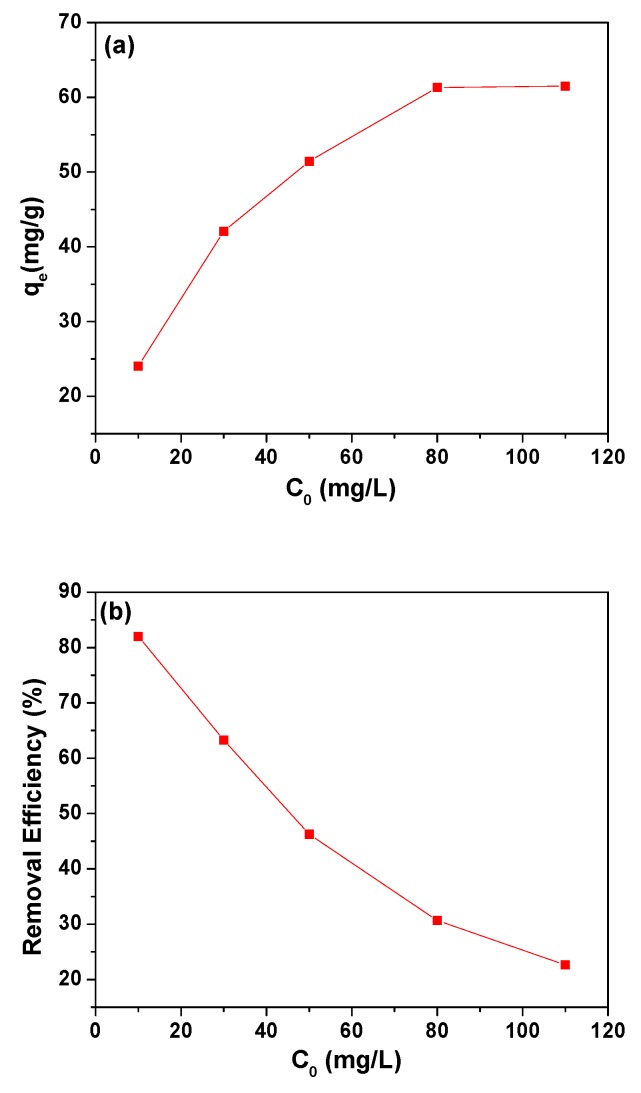
Effect of initial concentration of As(III) on (**a**) the equilibrium adsorption capacity and (**b**) removal efficiency of As(III) onto 3% MIL-53(Al)-GO (*M*/*V* = 0.4 g/L, pH = 6.1, and *T* = 298 K).

The effect of As(III) concentration (10–110 mg/L) on the adsorption of As(III) onto 3% MIL-53(Al)-GO was tested, and the results are plotted in Figure 5. An increase in the adsorption capacity of As(III) ions was observed with increasing the initial As(III) concentration from 10 to 80 mg/L, as presented in Figure 5a. This was due to the fact that with the increase of As(III) concentration, driving force required for mass transfer was increased. When As(III) ion concentration was increased, the increased concentration gradient acted as a driving force to overcome the resistance to mass transfer of the As(III) ions between the adsorbent and adsorbate species. Further increase in As(III) concentration (>80 mg/L) showed no significant change in equilibrium adsorption capacity. This occurred because the active sites for As(III) adsorption have been occupied, thereby showing almost no change in adsorption capacity [65].

Removal efficiency of As(III) in 3% MIL-53(Al)-GO was calculated using Equation (2). As shown in Figure 5b, removal efficiency of As(III) ions decreased with increasing the initial As(III) concentration. It is known that the removal efficiency of an adsorbate in an adsorption process is inversely related to the ratio of the number of adsorbate moieties to the number of the available active sites on an adsorbent (R_adsorbate/adsorbent_). R_adsorbate/adsorbent_ increases with increasing in the number of adsorbate moieties per unit volume of solution given a constant dosage of adsorbent [66]. The higher the value of R_adsorbate/adsorbent_ (i.e., the higher the surface coverage of an adsorbent), the lower the removal efficiency. Therefore, when the dosage of 3% MIL-53(Al)-GO remained constant, R_adsorbate/adsorbent_ increased with the increase in initial concentration if As(III), which resulted in the decrease in removal efficiency.

### 3.6. Effect of Adsorbent Dosage on As(III) Adsorption

Dosage of MOF-GO nanocomposites, that is the concentration of MOF-GO nanocomposites in heavy metal solution, showed a significant influence on As(III) adsorption. Dosage of 3% MIL-53(Al)-GO in As(III) solution was varied from 0.2 to 3 g/L. The effect of the dosage of 3% MIL-53(Al)-GO on the equilibrium adsorption capacity of As(III) ions is shown in Figure S3a. A decrease in As(III) adsorption was observed with increasing the dosage of 3% MIL-53(Al)-GO. Nevertheless, an opposite tendency was observed in As(III) removal efficiency, as presented in Figure S3b, where the As(III) removal efficiency was significantly increased with an increase in the adsorbent concentration from 0.2 to 1.6 g/L. As discussed earlier, R_adsorbate/adsorbent_ is related to the surface coverage of an adsorbent, which decreases with the increase in the dosage of an adsorbent at a constant initial concentration of the adsorbate. The smaller the value of R_adsorbate/adsorbent_, the more adsorption sites of the adsorbent for As(III) adsorption, the higher the removal efficiency. Therefore, removal efficiency of As(III) increased with the increase in dosage of 3% MIL-53(Al)-GO at a constant initial concentration of As(III) solution. It is noted that the removal efficiency reached a plateau with a maximum value of 94.8% when the dosage of 3% MIL-53(Al)-GO was beyond 1.6 g/L.

In general, MIL-53(Al)-GO nanocomposites can be regenerated using methanol, ethanol or ethylenediaminetetraacetic acid (EDTA). Studies have shown that the regenerated MOF-based nanocomposites still show good adsorption capacity of contaminants [48,67], indicating that MIL-53(Al)-GO nanocomposites could be promising and cost-effective adsorbents for water treatment.

## 4. Conclusions

In this work, a new metal-organic framework-graphene oxide nanocomposite, MIL-53(Al)-GO, was synthesized. It was demonstrated that the textural properties of the newly formed MIL-53(Al)-GO can be tailored by tuning the mass ratio of GO to MIL-53(Al); more importantly, MIL-53(Al)-GO exhibited higher surface area and higher adsorption capacity for As(III) compared to individual moieties and other traditional adsorbents (e.g., activated carbon, iron oxides, coconut husk; Table S1). A relatively fast (<1 h) and thermodynamically favorable adsorption of As(III) on MIL-53(Al)-GO nanocomposite was demonstrated. Optimal ratio composition of MIL-53(Al)-GO nanocomposite as well as optimal pH range for removal of As(III) were suggested in this study. Detailed kinetic and adsorption data reported here provides mechanistic insights into the As(III) adsorption on the MIL-53(Al)-GO nanocomposite. Overall, this is one of the first studies presenting the application a MOF-GO nanocomposite for heavy metal removal from aqueous solutions. This work opens new avenues for creating high surface area and high adsorption capacity MOF-based nanocomposite adsorbents for heavy metal removal in various water treatment/reuse applications.

## Figures and Tables

**Table 1:** Adsorption kinetics parameters of As(III) adsorption on GO, MIL-53(Al) and MIL-53(Al)-GO nanocomposites.

Model	Parameters	GO	MIL-53(Al)	1% MIL-53(Al)-GO	3% MIL-53(Al)-GO	15% MIL-53(Al)-GO	25% MIL-53(Al)-GO
Pseudo-first-order	*q_e,exp_* (mg/g)	2.05	32.65	49.73	51.80	32.45	37.55
	*k*_1_ (1/h)	0.18	0.23	0.18	0.26	0.08	0.23
	*q_e,cal_* (mg/g)	0.17	1.70	4.57	3.53	9.29	9.39
	*R* ^2^	0.323	0.289	0.311	0.447	0.345	0.561
Pseudo-second-order	*k*_2_ (g/mg·h^−1^)	14.19	1.40	0.73	0.62	0.28	0.26
	*q_e,cal_* (mg/g)	2.04	32.64	49.31	51.84	30.14	37.34
	*R* ^2^	0.999	0.999	0.998	0.999	0.996	0.998
Intra-particle diffusion	*K*_1*d*_ (mg/(g·h^1/2^))	0.48	17.03	21.54	21.31	20.79	21.31
	*C*_1_	1.63	17.04	28.26	30.54	9.37	16.29
	(*R*_1_)^2^	0.768	0.842	0.990	0.989	0.988	0.989
	*K*_2*d*_ (mg/(g·h^1/2^))	0.01	0.09	0.08	0.10	0.07	0.14
	*C*_2_	1.99	32.28	48.78	51.32	29.37	36.22
	(*R*_2_)^2^	0.227	0.441	0.035	0.267	0.012	0.028

**Table 2:** Isotherm parameters of As(III) adsorption on MIL-53(Al), GO, and 3% MIL-53(Al)-GO nanocomposite.

Adsorbent	Temperature (K)	Langmuir				Freundlich		
		*q_max_* (mg/g)	*K_L_* (L/mg)	*R_L_*	*R* ^2^	1/*n*	*K_F_* (mg/g)·(L/mg)^1/n^	*R* ^2^
GO	298	2.72	0.06	0.22	0.988	0.28	0.65	0.960
	308	2.12	0.07	0.19	0.988	0.26	0.56	0.971
	318	1.56	0.08	0.18	0.998	0.25	0.43	0.993
MIL-53(Al)	298	53.16	0.06	0.22	0.981	0.40	7.82	0.877
	308	59.21	0.05	0.26	0.991	0.44	7.36	0.943
	318	69.39	0.05	0.26	0.996	0.48	6.92	0.959
3% MIL-53(Al)-GO	298	64.97	0.24	0.07	0.999	0.26	21.08	0.944
	308	70.77	0.24	0.07	0.996	0.28	21.45	0.938
	318	78.55	0.20	0.08	0.999	0.29	22.06	0.949

## Supplementary Materials

The following are available online at http://www.mdpi.com/2079-4991/8/12/1062/s1, Figure S1: (a) XRD patterns and (b) FT-IR spectra of MIL-53(Al), GO and MIL-53(Al)-GO nanocomposites; Figure S2: SEM images of (a) GO, (b, c) MIL-53(Al), (d) 3% MIL-53(Al)-GO (e) 15% MIL-53(Al)-GO and (f) 25% MIL-53(Al)-GO.; Figure S3: The effect of 3% MIL-53(Al)-GO dosage on (a) the equilibrium adsorption capacity and (b) removal efficiency of As(III) ions (*C*_0_ = 50 mg/L, pH = 6.1 and T = 298 K); Table S1: List of some typical adsorbents for adsorbing As(III) under ambient pressure (*q_max_* = maximum adsorption capacity); Table S2: Thermodynamic parameters for As(III) adsorption on GO, MIL-53(Al) and 3% MIL-53(Al)-GO nanocomposite.
