# Supplementary material for: Removal of Arsenic(III) from Aqueous Solution Using Metal Organic Framework-Graphene Oxide Nanocomposite
Source: Nanomaterials (Basel). 2018 Dec 16;8(12):1062. doi: 10.3390/nano8121062 (PMC6315412; doi:10.3390/nano8121062)
Supplement: Supplementary file 1 [file nanomaterials-08-01062-s001.pdf]

Supplementary Materials for

**Removal of Arsenic(III) from Aqueous Solution Using Metal Organic Framework-Graphene Oxide Nanocomposite**

Tonoy Chowdhury[a], Lei Zhang[a]*, Junqing Zhang[a]*, Srijan Aggarwal[b]

[a]Department of Mechanical Engineering, PO Box 755905, University of Alaska Fairbanks, Fairbanks, AK 99775, USA

[b]Department of Civil & Environmental Engineering, PO Box 755900, University of Alaska Fairbanks, Fairbanks, AK 99775, USA

*Address correspondence to Lei Zhang, lzhang14@alaska.edu, Tel.:+1 907-474-6135, Fax: +1 907-474-6141; Junqing Zhang, jzhang16@alaska.edu, Tel.:+1 907-474-2647, Fax: +1 907-474-6141

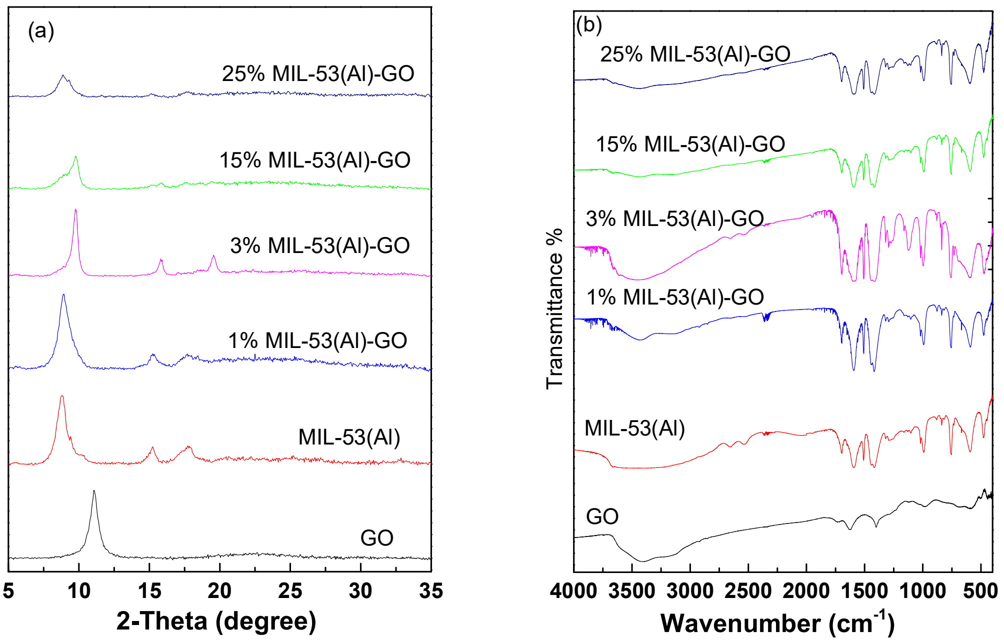


Figure S1 (a) XRD patterns and (b) FT-IR spectra of MIL-53(Al), GO, and MIL-53(Al)-GO nanocomposites.

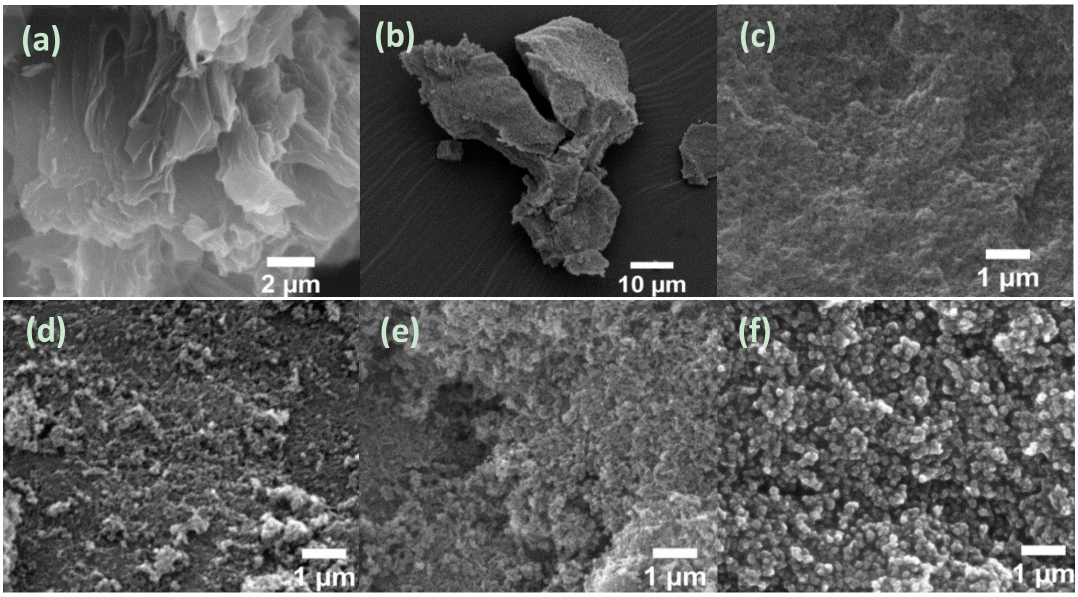


Figure S2 SEM images of (a) GO, (b, c) MIL-53(Al), (d) 3% MIL-53(Al)-GO, (e) 15% MIL-53(Al)-GO, and (f) 25% MIL-53(Al)-GO.

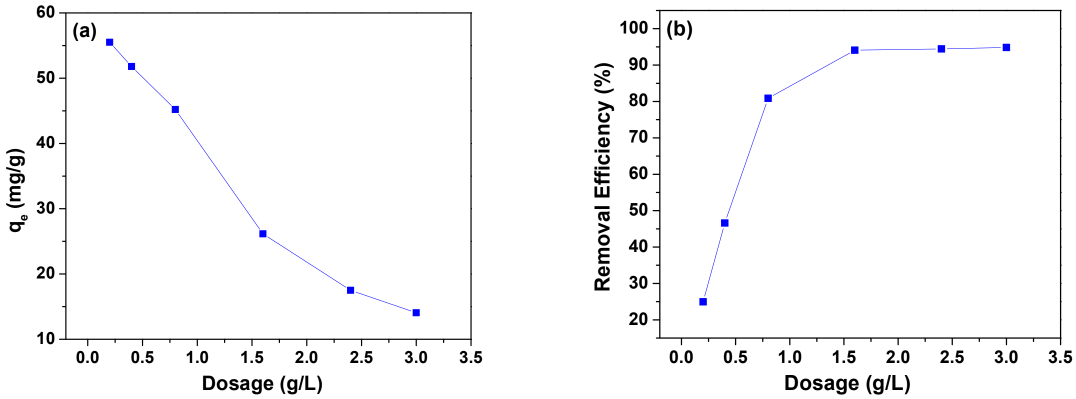


Figure S3 The effect of 3% MIL-53(Al)-GO dosage on (a) the equilibrium adsorption capacity and (b) removal efficiency of As(III) ions ($C_0$ = 50 mg/L, pH = 6.1, and $T$ = 298 K).

Table S1 List of some typical adsorbents for adsorbing As(III) under ambient pressure ($q_{max}$ = maximum adsorption capacity).

| Adsorbent | Optimum pH | Temperature (˚ C) | Adsorbent Dosage (g/L) | Surface Area ($m^2$/g) | $q_{max}$ (mg/g) | Ref. |
|---|---|---|---|---|---|---|
| Copper-impregnated coconut husk carbon | 6.5 | 30 | 2.0 | 206 | 20.35 | [1] |
| Amorphous iron hydroxide | 6.0-8.0 | - | 1.6 | - | 28.0 | [2] |
| Granular titanium dioxide | 7.0 | - | 1.0 | 250.7 | 32.4 | [3] |
| Iron-modified activated carbon | 7.6-8.0 | 20-23 | 0.1-20 | 723 | 38.8 | [4] |
| $Fe_2O_3$ nanoparticles | 7.0 | - | 0.06 | 178.48 | 46.06 | [5] |
| Gamma-$Fe_2O_3$ nanoparticles derived from MIL-100(Fe) | - | 25 | - | 1800 | 62.9 | [6] |
| 3% MIL-53(Al)-GO | 6.1 | 25 | 0.4 | 1147 | 64.97 | This work |

Table S2 Thermodynamic parameters for As(III) adsorption on GO, MIL-53(Al), and 3% MIL-53(Al)-GO nanocomposite.

| Adsorbent | $T$ (K) | $K_0$ | $\Delta G^0$(kJ/mol) | $\Delta S^0$(J/K.mol) | $\Delta H^0$(kJ/mol) | $R^2$ |
|---|---|---|---|---|---|---|
| GO | 298 | 0.12 | 5.25 | -89 | -21.35 | 0.953 |
| | 308 | 0.10 | 5.90 | | | |
| | 318 | 0.70 | 7.03 | | | |
| MIL-53(Al) | 298 | 1.49 | -0.99 | 33 | 8.91 | 0.985 |
| | 308 | 1.63 | -1.25 | | | |
| | 318 | 1.86 | -1.64 | | | |
| 3% MIL-53(Al)-GO | 298 | 3.92 | -3.38 | 13 | 0.49 | 0.996 |
| | 308 | 3.95 | -3.52 | | | |
| | 318 | 3.96 | -3.64 | | | |

**References**


1 Manju, G.N.; Raji, C.; Anirudhan, T.S. Evaluation of coconut husk carbon for the removal of arsenic from water. *Water Res.* **1998**, *32*, 3062-3070, doi:org/10.1016/S0043-1354(98)00068-2.

2 Lenoble, V.; Bouras, O.; Deluchat, V.; Serpaud, B.; Bollinger, J.-C. Arsenic adsorption onto pillared clays and iron oxides. *J. Colloid Interf. Sci.* **2002**, *255*, 52-58, doi:org/10.1006/jcis.2002.8646.

3 Bang, S.; Patel, M.; Lippincott, L.; Meng, X. Removal of arsenic from groundwater by granular titanium dioxide adsorbent. *Chemosphere* **2005**, *60*, 389-397, doi:org/10.1016/j.chemosphere.2004.12.008.

4 Chen, W.; Parette, R.; Zou, J.; Cannon, F.S.; Dempsey, B.A. Arsenic removal by iron-modified activated carbon. *Water Res.* **2007**, *41*, 1851-1858, doi:org/10.1016/j.watres.2007.01.052.

5 Feng, L.; Cao, M.; Ma, X.; Zhu, Y.; Hu, C. Superparamagnetic high-surface-area $Fe_3O_4$ nanoparticles as adsorbents for arsenic removal. *J. Hazard Mater.* **2012**, *217-218*, 439-446, doi:org/10.1016/j.jhazmat.2012.03.073.

6 Hei, S.T.; Jin, Y.; Zhang, F.M. Fabrication of gamma-$Fe_2O_3$ nanoparticles by solid-state thermolysis of a metal-organic framework, MIL-100(Fe), for heavy metal ions removal. *J. Chem.* **2014**, 6, doi:org/10.1155/2014/546956.
